# High-Quality Video Watermarking Based on Deep Neural Networks and Adjustable Subsquares Properties Algorithm

**DOI:** 10.3390/s22145376

**Published:** 2022-07-19

**Authors:** Maciej Kaczyński, Zbigniew Piotrowski

**Affiliations:** Faculty of Electronics, Military University of Technology, 00-908 Warsaw, Poland; maciej.kaczynski@protonmail.com

**Keywords:** neural network, watermark, deep learning, property verification, copyright protection, video, HEVC, H.265

## Abstract

This paper presents a method of high-capacity and transparent watermarking based on the usage of deep neural networks with the adjustable subsquares properties algorithm to encode the data of a watermark in high-quality video using the H.265/HEVC (High-Efficiency Video Coding) codec. The aim of the article is to present a method of embedding a watermark in a video with HEVC codec compression by making changes in a video in a way that is not noticeable to the naked eye. The method presented here is characterised by focusing on ensuring the accuracy of the original image in relation to the watermarked image, providing the transparency of the embedded watermark, while ensuring its survival after compression by the HEVC codec. The article includes a presentation of the practical results of watermark embedding with a built-in variation mechanism of its capacity and resistance, thanks to the adjustable subsquares properties algorithm. The obtained PSNR (peak signal-to-noise ratio) results are at the level of 40 dB or better. There is the possibility of the complete recovery of a watermark from a single frame compressed in the CRF (constant rate factor) range of up to 16, resulting in a BER (bit error rate) equal to 0 for the received watermark.

## 1. Introduction

Currently, video material uploaded to an Internet service can be very easily and quickly downloaded and then published further without the knowledge and consent of the author or the rights’ holder. Of course, there are many types and cases of copyright infringement; however, the description of this phenomenon is not the purpose of this publication. In view of this phenomenon, the protection of intellectual property in the digital field is an important aspect of today’s social life. To secure the rights to a video, a visible watermark is applied to the video for subsequent identification [1,2]. Despite the simplicity and effectiveness of this solution, the method has the significant disadvantage of leaving a visible mark on the entire video, which may affect its reception by the viewer of the video, and it also informs a person who is potentially interested in the unauthorised downloading and publication of the video about the security of the video. Potential infringers will not be discouraged by visible watermark information as there are methods available for the removal of visible watermarks. Due to the above-mentioned drawbacks of the solution for the protection of intellectual rights with a visible watermark, a watermark transparent to the human eye has been introduced, which leaves the reception by the target viewer undisturbed by redundant graphic information. Moreover, a potential copyright infringer may not be aware of the fact that the video material is protected [3,4]. However, it should be acknowledged that there are specialised methods designed to detect transparently embedded watermarks [5,6]; moreover, there are methods for attempting to remove such watermarks [7,8].

With the development of the issue of embedding a transparent watermark, there has been a demand for the development of new ways of embedding a watermark in a video. There are different methods for embedding a watermark in a video based on conventional methods, such as the discrete wavelet transform [9], the use of the least significant bit [10], and others mentioned later in this article; however, this article presents an approach that uses deep neural networks (DNNs) for this task. The methods outlined in the previous sentence are designed to embed a transparent watermark with the greatest possible capacity and resistance to distortion.

There are many papers in the literature dealing with video watermarking using artificial neural networks (ANNs) [11,12]. Among the works describing the process of embedding a watermark in a video, there are works dedicated specifically to a particular video codec, such as H.264/AVC (Advanced Video Coding) [13,14] and its successor H.265/HEVC (High-Efficiency Video Coding) [15,16].

Methods based on the use of ANNs, as well as classical methods, have their advantages and disadvantages in their applications, and they have the potential to be further improved. In this paper, the solution is based on the use of DNNs because neural networks are able to predict and find features of the problem under study that a creator of handwritten algorithms cannot predict; therefore, algorithms operating on deep networks are better-suited to solving various, highly complex problems. The necessary condition for such a statement is the assumption that a properly designed neural network architecture is selected and that it has an optimal training set.

The aim of this article is to introduce a robust watermarking method based on the use of DNNs with the adjustable subsquares properties encoding data algorithm dedicated to high-quality video compressed by HEVC codec. The main problem to overcome is assuring the survival of the watermark when the embedded frames are passed through the HEVC codec compression channel. It is a demanding task to embed a watermark and provide its resistance to compression channels in a way that does not degrade the visual quality of the video. As mentioned before, there are various approaches to solving this issue in the literature, and this paper presents a new approach based on a DNN autoencoder (with a compression channel between the encoder and the decoder during the learning process) and an original encoding watermark algorithm.

This paper is structured as follows: At the beginning of the paper, a literature review is presented. Then, the idea of the proposed method will be presented, along with a discussion of the adjustable subsquares properties algorithm. The learning process will be presented next, and the structures of ANNs will be demonstrated. Next, the results of the presented method will be discussed, together with their analysis and comparisons with other methods. The last section concludes this publication.

## 2. Literature Review

There are many works in the literature on the issue of embedding a watermark in an image. To generalize, they can be divided into classical methods and those using ANNs.

The classical methods include such methods as the least significant bit method [9], the mask method [17], and frequency-domain manipulations (discrete cosine transform [18], discrete sine transform [19], discrete wavelet transform [10], discrete Fourier transform [20], and hybrid domains [21,22,23], among others). Assorted variations of the above-mentioned methods are used to embed watermarks in both static images and videos.

In the literature, there are many publications on embedding a watermark in a static image using the classical methods [24,25,26,27,28,29,30,31,32,33,34]. These methods use the whole image or some of its regions, constituting the areas of interest. The least significant bit method [9,32], as its name implies, uses the least significant bit of each channel value of a given colour palette, affecting the image slightly, but it is prone to losing the watermark due to all kinds of conversions. The whole image is also influenced by methods that manipulate the frequency domain [18,19,20,21,22,23], which are characterized by better survival despite various types of conversions than are those generated via the method of the least significant bit. The modification of the most convenient areas takes place in different methods. An example of one such methods is that which allows for the selection of the optimal colour channel to be modified, as presented in the work of Huynh-The et al. [27].

With the development of ANNs and the areas of their use, they began to also be used to embed watermarks in static images [35,36,37,38]. The introduction of the use of ANNs has created new opportunities for research in watermark embedding, and those are also explored in this paper.

The subject of this publication is the embedding of a watermark in a video; therefore, it should be noted that there are various methods for this purpose, both classical [13,14,15,16,39,40,41,42,43] and those based on artificial neural networks [11,12]. Among the works describing the process of embedding a watermark in a video, there are some dedicated specifically to particular video codecs, such as H.264/AVC (Advanced Video Coding) [13,14,39,40,41] and its successor H.265/HEVC (High-Efficiency Video Coding) [15,16,42,43].

Embedding a watermark in a video is a more sophisticated issue than is that process with a static image due to the different characteristics of the codecs used to compress video material. For the purposes of embedding a video watermark, methods used with static images [12,13], as well as those dedicated to specific video codecs, are applied, using their specific properties [15,16,43]. Depending on the assumptions made, it is possible to recover the watermark from a single video frame or from a fixed number of frames.

In this article, a method based on using DNNs as applied to the HEVC codec to embed watermarks in videos will be discussed.

## 3. Proposed Method

### 3.1. Presentation of the Concept of the Proposed Method

The watermark embedding method proposed in this paper for H.265/HEVC (High-Efficiency Video Coding) encoded video is based on DNNs and an adjustable subsquares properties algorithm. The research was conducted on a Linux operating system using the TensorFlow version 2.7 machine learning library. The FFmpeg library was used to modify the video material, while the source codes were written in Python version 3.9.

**Figure 1:**
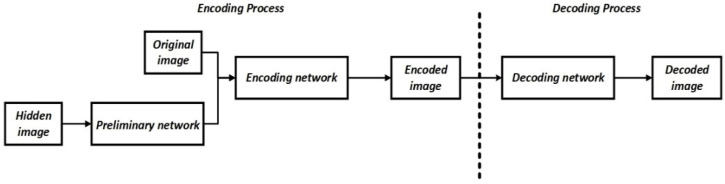
General conceptual diagram of the system components from the foundational article [44].

The following is the concept underlying the method, which is based on an article by Shumeet Baluja [44]. The article deals with hiding a static image in another static image. The method is based on the use of three ANNs (preliminary networks which prepare the hidden image, encode it, and decode it) as components that form a system for hiding the image and recovering it, the process for which is presented in Figure 1.

**Figure 2:**
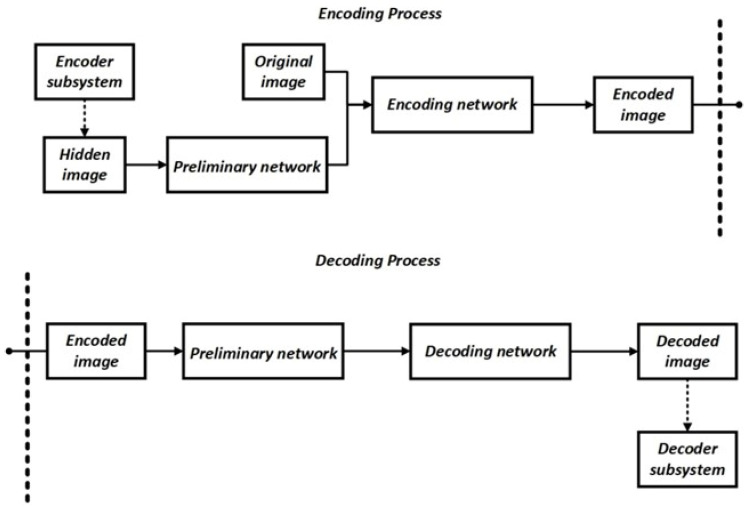
General conceptual diagram of the system components.

The method, presented hereafter, employs a modified version of the general system scheme, incorporating an additional preliminary network at the decoder input as well as at the encoder and decoder subsystems. The general conceptual scheme of the system components is shown in Figure 2; however, it is worth noting that this is a superficial representation of the system. The individual components presented in the diagram from the paper and in the diagram created from the actually conducted research are not the same, and they differ in their complexity.

At the very beginning, the encoder subsystem prepares a hidden image, which is a watermark, in accordance with the adjustable subsquares properties algorithm presented in the foundational article. Then, the hidden image is fed into the input of the preliminary network. The pre-prepared, hidden image, together with the original image, which is a carrier for the hidden image, is fed into the input of the encoding network. The encoder DNN substructure encodes the watermark in the carrier image by decomposing the secret image from the preliminary network to the set of features. The next step is to carry out the deconvolution of the obtained set of secret image features with the carrier image (achieved via the substructure of the encoder so as to achieve an encoded image carrier with the watermark) which embeds the watermark optimally so as to preserve the image as accurately as possible, as compared to the original image, while ensuring the possibility of recovering the watermark. During the learning process, the neural network automatically selects (learns) the optimal filters (of the convolutional layers) by which the image modification can be carried out in order to achieve the assumed goal.

The encoding of the data bits in the image is performed by encoding the bit data with the adjustable subsquares properties algorithm and obtaining a bit-encoded image. In the next step, the DNN encoder performs the optimal image encoding. The entire carrier image is subject to permanent modification because the watermark is embedded in the entire image area that is serviced by the encoder.

**Figure 3:**
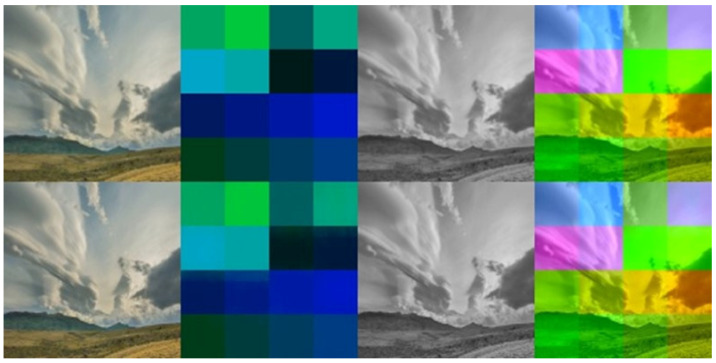
Preview of encoding effects: The top line of the figure shows the expected effect, while the bottom line shows the actual effect. The columns from left to right show the encoded image, the watermark converted later into a text form applied to the (UV) channels of the YUV colour palette, the preview of the Y luminance channel, and the preview of the whole image applied to the encoded image (all channels of the YUV colour palette).

After the image has passed through the compression channel of the HEVC codec, the decoding process begins. In the decoding process, the watermark image, which was created as a result of the encoding process, is fed into the input of the preliminary network of the decoder, and then the prepared image is fed into the input of the decoding network. The DNN decoder is responsible for the retrieval of the watermark from the carrier image. In the event that a watermark fails to embed in accordance with the proposed method, the recovered potential watermark will clearly return nonsensical, random information, and it will not be so clearly divided amongst the regions of interest according to the characteristics of the adjustable subsquares properties algorithm, as shown in Figure 3. The recovered watermark obtained at the output of the decoding network is processed by the decoder subsystem which, using the decoding algorithm mentioned in the previous sentence, identifies the watermark by recognizing the information encoded in the recovered image.

### 3.2. Adjustable Subsquares Properties Algorithm

To demonstrate the effect of the proposed method, the adjustable subsquares properties algorithm will be discussed first. The algorithm consists of dividing a square into a number of subsquares adequate to the specific size of the subsquare, chosen such that the sum of the sides of the subsquares in the square is equal to the side of the main square in which the subsquares are placed. In a variant of the method presented in this article, the size of the main square (hidden image) is assumed to be 128 × 128 pixels, and the size of the subsquare is assumed to be 32 × 32 pixels, making for a total of 16 subsquares. As digital images stored in popular colour palettes, such as RGB and YUV, have three channels of information, the main square also has three dimensions, such that the main square can be considered, in simple terms, to be a square image, with specific areas in it being smaller squares. Thus, by dividing the image in the above-described way, 16 areas of interest are obtained from an image with dimensions (128, 128, 3). At this point, it should be mentioned that the input and output of both the encoder and the decoder contain an image stored in the form of a YUV colour palette. Thus, the third dimension of the image, representing the individual channels gives three possible values to modify for each pixel; in the method under discussion, the modification of the two values of the U and V channels, representing chrominance, is adopted, while the Y channel, representing luminance, is not subject to modification.

The strength of the adjustable subsquare property manifests primarily in the adopted number system underlying the capacity of the system, together with the adopted size of the subsquare. Assuming that each channel is a representation of a digit in the adopted numeral system, it is possible to obtain as many as three digits in a given numeral system. For example, the maximum hidden value, starting from 0, for the binary numeral system is 7. For the senary numeral system, this value would be 215, and for the octal numeral system, this value would be 511. As mentioned previously, for the research presented in this paper, a two-channel variant was adopted; thus, referring to the examples in the previous sentence, the maximum values for each numeral system will change accordingly: For the binary numeral system, the maximum value will be 3; for the senary numeral system, 35; and for the octal numeral system, 63. For the purpose of presenting the method outlined in this paper, the senary numeral system has been adopted. A graphical depiction of the concept of the adjustable subsquares properties algorithm is shown in Figure 1, while the properties of the method are described by the following relationships:

(1) $$SteepValue_{min}=\frac{L}{RGB_{\max}}=\frac{25}{255}=0.09803921568$$

(2) $$SteepValue=\left(\frac{\left(\frac{B\;*\;M}{RGB_{max}}\right)}{\left(B*2\right)}\right)=\left(\frac{\left(\frac{5\;*\;35}{255}\right)}{\left(5*2\right)}\right)=0.06862745098$$

(3) $$SteepValue_{min\left[0;255\right]}=\frac{25}{255}*255=25$$

(4) $$SteepValue_{\left[0;255\right]}=\left(\frac{\left(\frac{5\;*\;35}{255}\right)}{\left(5*2\right)}\right)*255=17.5$$

(5) $$SteepValue_{final}=SteepValue_{min}+SteepValue*DigitMultiplier\;$$

where $RGB_{\max}$ is the maximum value in the RGB colour palette (255), L is the minimum value of the watermark embedding range in the value interval 〈0; 255〉, $SteepValue_{min}\;$is the minimum constant value added to the step calculating the value range for the digit, B is the maximum value of the digit of the used numeral system (senary), M is the maximum value of the range as a multiplier of the numeral system (senary) used, SteepValue is the constant value of the step calculating the value range for the digit, $SteepValue_{\left[0;255\right]}$ is the constant value of the step calculating the range of values for the digit in the RGB value range, $SteepValue_{min\left[0;255\right]}$ is the minimum value added to the step calculating the range of values for the digit in the RGB value range, DigitMultiplier is the multiplier for the digit specifying the lower- or upper-range value for the digit within the integer value range 〈0; B∗2〉, and $SteepValue_{final}$ is the total value of the step calculating the range of values for the digit.

The system’s basic purpose is to encode 16 ASCII characters using an image fragment of 128 × 128 pixels from the range of Arabic numerals (0–9) and the letters of the English alphabet (A-Z), making for a total of 36 possible ASCII characters. Before the character concealment operation is performed, the image undergoes a change from the RGB colour palette to the YUV colour palette and a normalisation from the number range 〈0; 255〉 to the floating-point number range in 〈0; 1〉. ASCII characters are encoded on a 128 × 128 pixel image in 32 × 32 pixel blocks. The U and V colour channels are used to store the characters. The Y channel, which is the luminance channel, is trained to represent the original Y channel. The characters are encoded in the U and V channels in the senary numeral system. The U channel contains the older bit, while the other channel contains the younger bit. The value of the digit is stored in the interval 〈0; 1〉 with the step value calculated according to the previously presented relations.

The value ranges on which the digits are encoded are shown in Table 1; the limiting value in the translation in the RGB colour palette value system is 182.5; it is the limiting value for digit 4; values greater than this will represent digit 5.

At this point, it is worth noting that, according to the system components presented, two ANNs are used in the encoding process. The first one prepares the image to be hidden in a form matching the input of the encoder, and the second one is the encoder, which receives at its input the appropriately transformed, hidden image and the original image in which it is to be embedded. The result of the encoder’s operation is an image which is supposed to be, for the human eye, indistinguishable from the original, but which has encoded information. After embedding the hidden image in the input image, the image, so prepared, can be fed into the input of the decoder. The decoder, just like the encoder, also has a network for processing the image. The decoder reads the hidden image from the image and then converts it into text form using the decoder subsystem. The higher the resolution of the image, the more watermark repetitions are read from a single frame. At 2160 × 3840 (UHD) resolution, a single frame results in 464 watermark repetitions measuring 128 × 128 pixels. All watermark repetitions are subjected to a median function operation that best represents the value of individual pixels for decoding.

An example of the results of the described system for watermarking video material is shown in Figure 3. The system user can encode the watermark stored as a text represented as ASCII characters from the range of Arabic numerals (0–9) and the letters of the English alphabet (A–Z), which is a total of 36 possible ASCII characters, or as a binary string of characters stored as shown in Table 2. The total number of characters for a 128 × 128 pixel encoder is 16 characters, or 80 bits. To avoid increasing the complexity of the system, and simultaneously the knowledge of the user, the characters whose encoding does not fit into 5 bits (V, W, X, Y, and Z) are not available when reading binary characters from a file, but they are available and work correctly when reading characters from a text file.

### 3.3. Learning Process

For the purpose of learning the deep SSNs constituting the encoder and decoder, as previously shown in Figure 2, the following relationships were applied for the purpose of adjusting the objective function accordingly:

(6) $$MSE_{EncImg}=\mathrm{MSE}\left({\mathrm{Image}}_{\mathrm{Original}},\;{\mathrm{Image}}_{\mathrm{Encoded}}\right)$$

(7) $$MSE_{DecImg}=\mathrm{MSE}\left({\mathrm{Image}}_{\mathrm{ToHide}},\;{\mathrm{Image}}_{\mathrm{DecodedHidden}}\right)$$

(8) $$MSE_{EncVid}=\mathrm{MSE}\left({\mathrm{Image}}_{\mathrm{Original}},\;{\mathrm{ImageFromVideo}}_{\mathrm{Encoded}}\right)$$

(9) $$MSE_{DecVid}=\mathrm{MSE}\left({\mathrm{Image}}_{\mathrm{ToHide}},\;{\mathrm{ImageFromVideo}}_{DecodedHidden}\right)$$

(10) EpochChange(i)=i∗0.001

(11) $$L_{EncLoss}\left(i\right)=\left(\left(\left(1.01-\mathrm{EpochChange}\left(i\right)\right)\;*\;MSE_{EncImg}\right)+\left(\left(0.50+\mathrm{EpochChange}\left(i\right)\right)\;*\;MSE_{EncVid}\right)\right)\;i\;\epsilon \;(0\;;\;500\rangle$$

(12) $$L_{DecLoss}\left(i\right)=\left(\left(\left(1.01-\mathrm{EpochChange}\left(i\right)\right)\;*\;MSE_{DecImg}\right)+\left(\left(0.50+\mathrm{EpochChange}\left(i\right)\right)\;*\;MSE_{DecVid}\right)\right)\;i\;\epsilon \;(0\;;\;500\rangle$$

(13) $$L_{EncLoss}\left(i\right)=\left(\left(\left(1.01-\mathrm{EpochChange}\left(i\right)\right)\;*\;MSE_{EncImg}\right)+\left(\;MSE_{EncVid}\right)\right)\;i\;\epsilon \;(500\;;\;1000\rangle$$

(14) $$L_{DecLoss}\left(i\right)=\left(\left(\left(1.01-\mathrm{EpochChange}\left(i\right)\right)\;*\;MSE_{DecImg}\right)+\left(MSE_{DecVid}\right)\right)\;i\;\epsilon \;(500\;;\;1000\rangle$$

(15) $$L_{EncLoss}\left(i\right)=\left(\left(0.01\;*\;MSE_{EncImg}\right)+\left(\;MSE_{EncVid}\right)\right)\;i>1000$$

(16) $$L_{DecLoss}\left(i\right)=\left(\left(0.01\;*\;MSE_{DecImg}\right)+\left(MSE_{DecVid}\right)\right)\;i>1000$$

(17) $$L_{EncFinalLoss}\left(i\right)=\left(L_{EncLoss}\left(i\right)+\left(0.56\;*\;L_{DecLoss}\left(i\right)\right)\right)$$

(18) $$\nabla L_{Enc}\left(i\right)=\frac{\partial L_{EncFinalLoss}\left(i\right)}{\partial Var_{\mathrm{Enc}}\left(i\right)}$$

(19) $$\nabla L_{Dec}\left(i\right)=\frac{\partial L_{DecLoss}\left(i\right)}{\partial Var_{\mathrm{Dec}}\left(i\right)}$$

where MSE is the mean square error; ${\mathrm{Image}}_{\mathrm{Original}}$ is the original image; ${\mathrm{Image}}_{\mathrm{Encoded}}$ is the image with the embedded watermark; ${\mathrm{Image}}_{\mathrm{ToHide}}$ is the image (watermark) hidden in the original image; ${\mathrm{Image}}_{DecodedHidden}$ is the hidden image recovered from the encoded image (with the embedded watermark); ${\mathrm{ImageFromVideo}}_{\mathrm{Encoded}}$ is the image from a single video frame; ${\mathrm{ImageFromVideo}}_{DecodedHidden}$ is the hidden image recovered from the encoded image (with the embedded watermark) from a single video frame; $MSE_{EncImg}$ is the mean square error of the original image vs. that of the encoded image; $MSE_{DecImg}$ is the mean square error of the hidden image vs. that of the decoded image; $MSE_{EncVid}$ is the mean square error of the original image vs. that of the encoded image in a single video frame; $MSE_{DecVid}$ is the mean square error of the hidden image vs. that of the image decoded from a single video frame; i is the learning epoch number; EpochChange(i) is a value modifying the error function calculated from the epoch number; $L_{EncLoss}\left(i\right)$ is the image-encoding (watermark-embedding) error; $L_{DecLoss}\left(i\right)$ is the decoding error (watermark recovery); $L_{EncFinalLoss}\left(i\right)$ is the summed error of the encoder and decoder, including the weights of the individual errors; $Var_{\mathrm{Enc}}\left(i\right)$ is the tensor of the encoder model weights; $Var_{\mathrm{Dec}}\left(i\right)$ is the tensor of the decoder model weights; is the gradient of the encoder’s objective function; and $L_{Dec}\left(i\right)$ is the gradient of the decoder’s objective function.

For the implementation of the learning process, the Adam Optimizer [45] was used, which is successfully applied to optimise multivariate objective function problems. The learning set consisted of 4000 randomly downloaded images from the Web, which were changed in resolution, according to the image size supported by the SSN, to 128 × 128 pixels. After conversion from the RGB to the YUV colour palette, these images were then directly used as ${\mathrm{Image}}_{\mathrm{Original}}$ in the training process, as well as for computing both *MSE_EncImg_* and $MSE_{EncVid}$. For the purpose of embedding the watermark in the video, the watermarked images of the learning set were also saved as video files in H.265 format, with the video compression ratio *CRF* = 7, and then read as ${\mathrm{ImageFromVideo}}_{\mathrm{Encoded}}$ for the purpose of computing $MSE_{EncVid}$. The hidden images ${\mathrm{Image}}_{\mathrm{ToHide}}$ were randomly generated in such a way that the data storage system imposed by the adjustable subsquares properties algorithm was preserved. The numerical values of the digits shown in Table 1 were drawn as the value of a digit was taken as the median value from the corresponding range of values. The test set, on the other hand, was composed of the individual $MSE_{DecVid}$ frames of the video, encoded and then read after HEVC recording.

The neural network structure described in this work is based in its assumption of the operation presented in the work previously mentioned at the beginning of this section [1]. The autoencoder structure consists of an encoder, which is a network that embeds the hidden watermark in the image, and a decoder, which returns the recovered hidden watermark as an output. Similar to the structure from the work mentioned at the beginning of this paragraph, where the input data is an RGB image which is a three-channel tensor, in the presented method, the input data is a YUV image which is also a three-channel tensor. Table 3, Table 4 and Table 5 show the parameters of the DNN encoder and decoder structures. As shown in Figure 2, the proposed method includes encoding, decoding, and preliminary networks (which are identical in their structure for the needs of the encoder and decoder, but, of course, each of them has been trained for the needs of the encoder or decoder, respectively).

The preliminary networks for the encoder and decoder are identical in their structure, but they have different roles. For the encoder, the preliminary network processes the original image so that the encoding network can embed the watermark with better results, while, for the decoder, the preliminary network prepares the output image for the decoding network. The network structure includes the following layers: Convolution 2D, Batch Normalization, and Concatenate. The LeakyReLU activation function is used for the convoluted layers. At the input of the encoding network, a tensor—combined from the image processed by the preliminary network and the watermark—is applied, while the output of the network is expected to be the image with the embedded watermark. The decoding network receives at its input a pre-processed image in the form of a tensor from the preliminary network, which it further processes and returns as an embedded watermark at its output.

The preliminary networks for the encoder and decoder are identical in structure. The encoding and decoding networks have similar network structures, the difference being the number of filters used.

Memory and performance requirements for processing a single 128 × 128 pixel image are shown in Table 6.

### 3.4. Edge Effect

**Figure 4:**
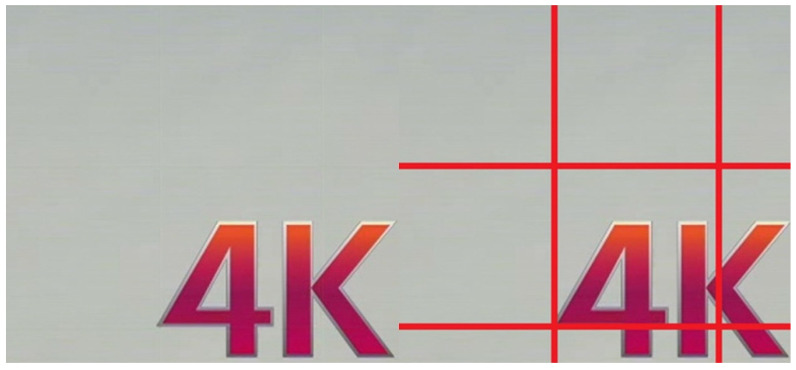
Preview of the edge effect: edge effect on the left; selection of the occurrence of an edge effect on the right.

When embedding a watermark into an image larger than the input size of 128 × 128 pixels, an edge effect is noticeable, consisting of a distortion of a few pixels at the edges of the encoded portion. The distortion is visible when zoomed in appropriately, depending on the size of the image. An example of an edge effect is shown in Figure 4.

The effect of edge distortion is offset by additional computations involving the embedding of watermarked image chunks (which are the edges of the encoded chunks) by moving them appropriately on the original image so that the encoder window contains within it the chunks of the original image that were previously on the edges of the encoder. Combining the result of the encoded image using the simple, sliding window technique and the results obtained from the original image to compensate for the edge effect creates an output image without visible distortion.

## 4. Results

### 4.1. Results of the Research

**Figure 5:**
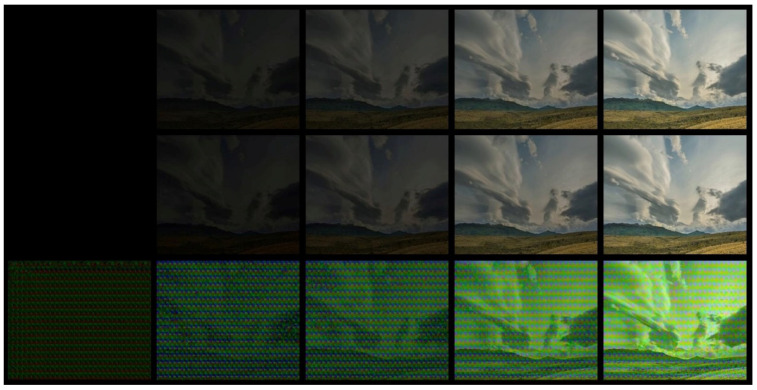
Preview of ANN results: From top-left to bottom: original frame, encoded frame, decoded frame) for Frame 1, 6, 9, 20, and 30 and CRF 0.

**Figure 6:**
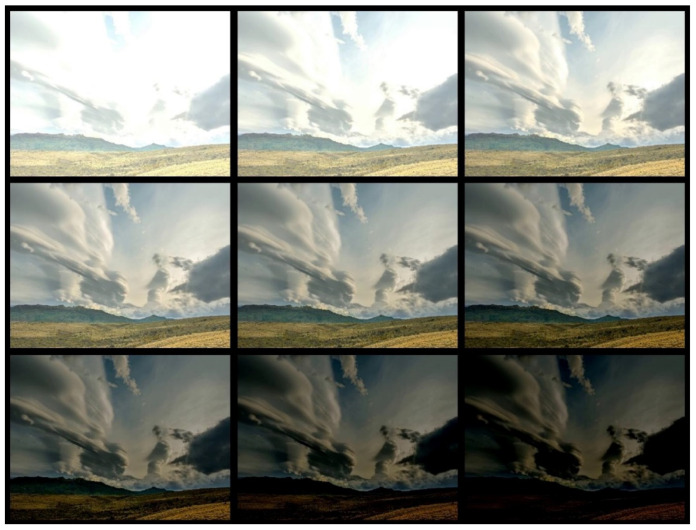
Preview of the brightness factor change for the 30th frame: From top-left: BF 4, BF 3, BF 2, BF 1, BF 0, BF −1, BF −2, BF −3, and BF −4.

**Figure 7:**
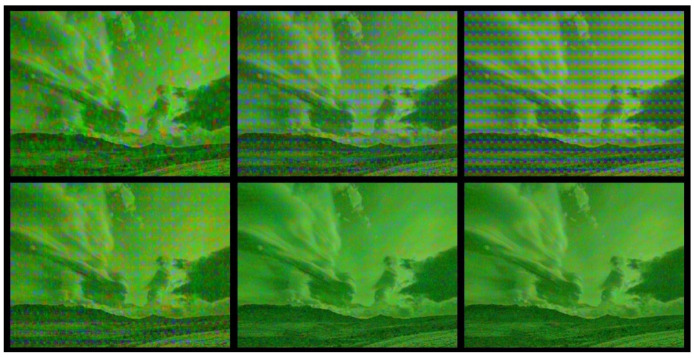
Preview of decoding results by ANN: From top-left, resolutions of 1080 × 1920, 2032 × 3712, 2160 × 3840, 2288 × 3968, 4320 × 7680, and 4608 × 8192 for the 21st frame and CRF 7.

This section presents the results of the video watermarking system when the video compression rate is changed, which is represented by the CRF (constant rate factor) (Table 7 and Table 8; Figure 5) and when changing the video BF (brightness factor) (Table 9; Figure 6). The impact of changing the video resolution will also be discussed (Table 10 and Table 11; Figure 7). For the purpose of testing the effectiveness of the presented method, an embedded watermark in the form of the string “QVERTS0123456789” was adopted. The test-video fragment for the presentation of the method in this paper contained 30 frames of video footage of moving objects in the form of clouds visible in the image, and it was characterised by a gradual brightening, starting from a complete darkening of the image [46]. Example video frames are shown in Figure 5.

According to the results shown in Table 7, there is an apparent trend in the overall retrieval of the watermark from the individual video frames for the CRF in the range 〈0; 16〉. According to the results in the table for CRF values greater than 17, the recovered mark is partially degraded as the compression ratio value increases. Analysing the results in Table 8, there is a trend in the similarity of the original image to the encoded image, measured by MSE to 5 decimal places. In contrast with the well-known fact in steganography that it is easier to hide information in a brightened image, this method has no need to do so, because embedding a watermark slightly affects the original image, as shown in the results in the table.

The effect of changing the brightness, both by brightening and darkening, is shown in Table 9. As the results of the brightness-change study show, the presented method shows better resistance to image brightening than to image darkening. Additionally, it is worth noting that, for a darkened image, changing the brightness of the image does not degrade the watermark to the extent that it cannot be recovered. However, for very dark backgrounds which are almost black or black, when the image brightness is changed, the watermark is lost in these areas. A preview of the brightness factor changes is shown in Figure 6, and a preview of the original frames is shown in Figure 5.

Changing the resolution negatively affects the embedded watermark. The presentations of the results of the resolution changes in Table 10 and Table 11 show the results for changes from the UHD resolution of the images to another resolution. A preview of the decoding results after the resolution changes is shown in Figure 7.

**Figure 8:**
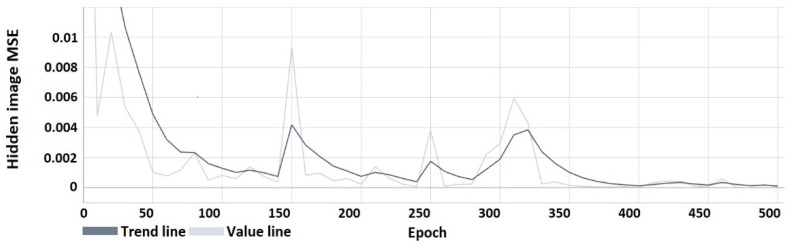
Mean squared error of the hidden image relative to the recovered image.

**Figure 9:**
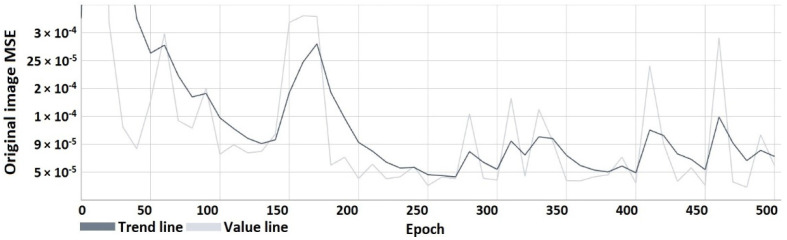
Mean squared error of the watermarked image relative to the original image.

**Figure 10:**
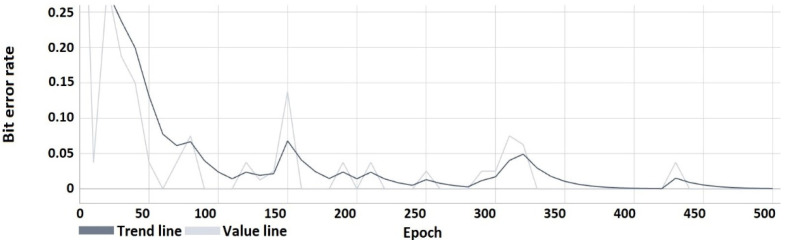
Bit error rate of the hidden message.

**Figure 11:**
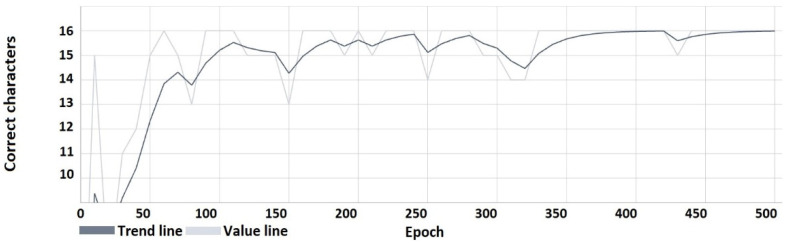
The absolute accuracy of the hidden message (number of correctly recovered characters).

The following characteristics (Figure 8, Figure 9, Figure 10 and Figure 11) show the results obtained during the learning process. From the course of the characteristics, the trend towards correct watermark recovery is noticeable, starting from the 330th learning epoch onwards, for which the BER is 0.

### 4.2. Comparison with Other Methods

In this subsection, the proposed method is compared with the selected state-of-the-art (SOTA) methods mentioned earlier in the literature review.

In order to make the comparison, the obtained MSE results were converted into the PSNR coefficient according to the following formula [47]:

(20) $$\mathrm{PSNR}=10\log_{10}\left(\frac{{\left(2^{M}-1\right)}^{2}}{\mathrm{MSE}}\right)$$

where PSNR stands for peak signal-to-noise ratio given in dB, M is the number of bits needed to define the range of values that a pixel can take, and MSE is an abbreviation for the mean square error.

It should be noted that the MSE given in the previous tables was calculated for the floating-point values at the encoder input and output because neural networks usually take values in the range 〈0; 1〉 due to the normalization process. In the comparison with other methods, these values will be compared with MSE values in the range 〈0; 255〉.

To ensure the comparability of the methods in the experiment, the selected compression value for HEVC is 16. The results of the method comparison are presented in Table 12.

Each method uses a different way to embed a watermark. The first method [15] presents an intra-drift-free watermarking algorithm, which uses a multi-coefficient modification method. This method embeds the watermark into intra prediction residual pixels of 4 × 4 luminance transform blocks in the spatial domain.

In the second method [16], initially, a spatial texture analysis is performed based on the number of non-zero transform coefficients of embedding blocks. Then, suitable candidate blocks for watermark embedding are selected. In the next step, the grouping of intra prediction modes is performed. Each group is represented by two bits of watermark sequence. The embedding process is performed by altering the prediction modes of selected 4 × 4 intra prediction blocks to the representative mode of the group denoted by the watermark bit pair.

The third method [43] uses the BCH syndrome code technique. In this method, groups of the prediction directions are provided to limit the intra-frame distortion drift. The encoded data are embedded into the multi-coefficients of the selected 4 × 4 luminance discrete sine transform blocks as required by pre-defined groups.

A detailed description of the proposed method of high-capacity and transparent watermarking based on the usage of DNNs with the adjustable subsquares properties algorithm is presented in Section 3.

The average PSNR for the proposed method and the compared methods is greater than 40 dB, which provides an acceptable visual experience.

The time needed to compute the proposed method is much greater compared to that required by the other methods; this is due to the fact that the ANN structure is not optimized for real-time processing. The currently proposed ANN structure is a research structure and is only applicable for offline watermark embedding. The embedding time for the proposed method also includes the time taken to remove the edge effect described in Section 3.4, which doubles the watermarking time. An additional factor that increased the computation time was the need to encode 8 areas of 128 × 128 in order to embed a watermark on the entire image with a size of 416 × 240. It is worth noting that watermark repeats were embedded, while other watermark combinations could be embedded by creating sequences of watermarks to compose one larger watermark. In order to accelerate the computation, the structures of the ANNs proposed in this paper should be reduced.

The compared methods [15,16,43] have a watermark capacity of 100 bits on the test sets, with the smallest image size being 416 × 240. However, the presented variant of the adjustable subsquares properties algorithm offers a capacity of 80 bits in a 128 × 128 image. By increasing the size of the encoded image with the proposed method to a size close to the test size for compared methods to the size of 224 × 224, the watermark capacity with the test variant of the adjustable subsquares properties algorithm increases to 245 bits. In accordance with the previous sentence, the proposed method has a greater watermark capacity than the compared methods. This capacity can be further increased by modifying the parameters of the adjustable subsquares properties algorithm.

## 5. Conclusions

The process of training DNNs for video watermark embedding is a computationally resource-intensive task. The main reason for this is the resolution at which the ANN operates during the training process. By increasing the resolution of the learning patterns, i.e., the resolution at which the network will operate natively, the hardware resource requirements increase significantly. Given this fact, it is reasonable to train ANNs (encoders and decoders) at lower resolutions and then, through an appropriate algorithm, transpose their operations to higher image resolutions. Such a solution facilitates the process of training and searching for the optimal structure of the neural network, but it also enables the encoding of arbitrary image resolutions. Additionally, such a solution allows for multiple encodings of the same watermark in a larger image (a single frame of video), thus facilitating the process of watermark recovery. Moreover, it is possible to create a scenario of a larger image that includes different watermarks which, when combined, form one larger watermark.

Encoding the watermark in the YUV colour palette seems to be characterised by a more permanent watermark deposition in the chrominance, i.e., in the U and V channels, than in the luminance Y channel. When designing a video watermarking system, it is worth bearing in mind the range of values in which the YUV and RGB colour palettes operate and, in particular, the fact that the YUV colour palette can also have negative values, while RGB has only positive values. This is not only important for the design of the watermark embedding system, but it is also crucial when using ANNs, forcing the selection of an appropriate activation function that takes negative values into account. The proper selection of the value range in which the watermark is embedded has a noticeable effect on the image in which it is hidden. When attempting to hide a white image in a black one (in the RGB colour palette, this would be hiding the 255 value in the 0 value), the network tends to learn to brighten the image so as to hide the watermark and keep the original image as true as possible, which results in an unwanted, visible brightening of the image. One possible solution to the above problem is to change the value range in which the watermark is embedded so as to reduce the size of the difference between the numerical values of each individual pixel of the original image and the watermark. Another approach is to attempt to apply an appropriate thresholding of the watermark energy, depending on the energy of the original image.

The method presented in this paper generates desirable results for high image quality (low video-compression ratio), that is CRF, in the range 〈0; 16〉 and demonstrates a tolerance for changes in image brightness—less for a darkened image, and more for a brightened one. Changing the encoded video to a different resolution results in a degradation of the watermark—greater for reduced video resolution and less for increased video resolution.

The issues of changing the resolution of a watermarked video and embedding a watermark for a higher video compression ratio require further research. The question of increasing the capacity of the watermark and reducing the structure of the ANNs to speed up the method also requires further exploration.

In order to reduce the structure of the neural network, the number of filters can be reduced. To make the watermark more resistant to image-resolution changes, it is worth introducing an additional component improving the quality of the recovered watermark, such as a super-resolution neural network. Increasing the capacity of the watermark can be achieved by appropriately selecting the parameters of the adjustable subsquares properties algorithm. Improving the codec compression tolerance for correct watermark embedding can be achieved by introducing an additional neural network sub-structure to show the learning neural network the results of higher HEVC codec compression and its effect on the hidden watermark.

## Figures and Tables

**Table 1:** Value ranges for encoding and decoding digits.

Digit	Value Range	RGB Value Range
0	〈0; 0.16666666666〉	〈0; 42.5〉
1	(0.16666666666; 0.30392156862〉	(42.5; 77.5〉
2	(0.30392156862; 0.44117647058〉	(77.5; 112.5〉
3	(0.44117647058; 0.57843137254〉	(112.5; 147.5〉
4	(0.57843137254; 0.71568627451〉	(147.5; 182.5〉
5	(0.715686274515; 1〉	(182.5; 255〉

**Table 2:** Value ranges for encoding and decoding digits.

No.	Character	Binary Representation	No.	Character	Binary Representation
1	0	00000	19	I	10010
2	1	00001	20	J	10011
3	2	00001	21	K	10011
4	3	00011	22	L	10100
5	4	00100	23	M	10101
6	5	00101	24	N	10110
7	6	00110	25	O	10111
8	7	00111	26	P	11000
9	8	01000	27	Q	11001
10	9	01001	28	R	11010
11	A	01010	29	S	11101
12	B	01011	30	T	11110
13	C	01100	31	U	11111
14	D	01101	32	V	
15	E	01110	33	W	
16	F	01111	34	X	
17	G	10000	35	Y	
18	H	10001	36	Z	

**Table 3:** Basic Layer (BL) processing model.

Layer Number	Layer Type
1	Convolution 2D layer
2	Batch Normalization
3	Convolution 2D layer
4	Batch Normalization
5	Convolution 2D layer
6	Batch Normalization

**Table 4:** Preliminary, convolutional neural network model (for encoder and decoder).

Layer Number	Layer Type	Parameters
1	BL	activation function: LeakyReLU kernel size: 3 × 3 filter number: 61
2	BL	activation function: LeakyReLU kernel size: 5 × 5 filter number: 61
3	BL	activation function: LeakyReLU kernel size: 7 × 7 filter number: 61
4	Concatenate ((1, 2, 3), axis = 3)	-
5	BL	activation function: LeakyReLU kernel size: 7 × 7 filter number: 61
6	BL	activation function: LeakyReLU kernel size: 5 × 5 filter number: 61
7	BL	activation function: LeakyReLU kernel size: 3 × 3 filter number: 61
8	Concatenate ((5, 6, 7), axis = 3)	-

**Table 5:** A model of convolutional neural network encoding and decoding.

Layer Number	Layer Type	Parameters
1	BL	activation function: LeakyReLU kernel size: 3 × 3 encoder filter number: 61 decoder filter number: 71
2	BL	activation function: LeakyReLU kernel size: 5 × 5 encoder filter number: 61 decoder filter number: 71
3	BL	activation function: LeakyReLU kernel size: 7 × 7 encoder filter number: 61 decoder filter number: 71
4	Concatenate ((1, 2, 3), axis = 3)	-
5	BL	activation function: LeakyReLU kernel size: 7 × 7 encoder filter number: 61 decoder filter number: 71
6	BL	activation function: LeakyReLU kernel size: 5 × 5 encoder filter number: 61 decoder filter number: 71
7	BL	activation function: LeakyReLU kernel size: 3 × 3 encoder filter number: 61 decoder filter number: 71
8	Concatenate ((5, 6, 7), axis = 3)	-
9	Convolution 2D layer	activation function: LeakyReLU kernel size: 1

**Table 6:** Parameters of the designed networks.

Type	Input Tensor Size	Number of Weights	Size on Disk	Processing Time for a Single Tensor [ms]	GPU Processor
Encoder	(2, 128, 128, 3)	6,523,953	75.3 MB	106.681	GeForce 1080Ti GTX 11GB
Decoder	(128, 128, 3)	7,647,390	88.2 MB	31.25	

**Table 7:** Algorithm performance results for a 2160 × 3840 encoded video fragment with a 128 × 128 encoder trained for CRF 7 when changing the CRF-16-character-string decoding representation.

Frame #	CRF 0	CRF 1	CRF 4	CRF 7	CRF 10	CRF 13	CRF 16	CRF 19	CRF 22	CRF 23
1	S ^1^: 4	S: 4	S: 4	S: 3	S: 2	S: 3	S: 3	S: 2	S: 1	S: 1
	M ^2^: 4	M: 4	M: 4	M: 3	M: 2	M: 3	M: 3	M: 2	M: 1	M: 1
5	S: 16	S: 16	S: 16	S: 16	S: 16	S: 16	S: 13	S: 10	S: 3	S: 3
	M: 16	M: 16	M: 16	M: 16	M: 16	M: 16	M: 15	M: 12	M: 6	M: 3
10	S: 16	S: 16	S: 16	S: 16	S: 16	S: 16	S: 16	S: 13	S: 4	S: 2
	M: 16	M: 16	M: 16	M: 16	M: 16	M: 16	M: 16	M: 11	M: 4	M: 3
15	S: 16	S: 16	S: 16	S: 16	S: 16	S: 16	S: 16	S: 12	S: 2	S: 2
	M: 16	M: 16	M: 16	M: 16	M: 16	M: 16	M: 16	M: 12	M: 4	M: 2
20	S: 16	S: 16	S: 16	S: 16	S: 16	S: 16	S: 16	S: 14	S: 2	S: 2
	M: 16	M: 16	M: 16	M: 16	M: 16	M: 16	M: 16	M: 12	M: 3	M: 2
25	S: 16	S: 16	S: 16	S: 16	S: 16	S: 16	S: 14	S: 6	S: 2	S: 2
	M: 16	M: 16	M: 16	M: 16	M: 16	M: 16	M: 16	M: 12	M: 3	M: 2
30	S: 16	S: 16	S: 16	S: 16	S: 16	S: 16	S: 16	S: 11	S: 2	S: 2
	M: 16	M: 16	M: 16	M: 16	M: 16	M: 16	M: 16	M: 11	M: 2	M: 2

^1^ Single frame containing subsquares, ^2^ Median of the decoded values of the sum of all frames.

**Table 8:** Algorithm performance results for a 2160 × 3840 encoded video fragment with a 128 × 128 encoder trained for CRF 7 when changing the CRF representation of the decoding (16 characters corresponding to 80 bits in the adopted numerical notion) in the forms of BER and MSE.

Frame #	CRF 0	CRF 1	CRF 4	CRF 7	CRF 10	CRF 13	CRF 16	CRF 19	CRF 22	CRF 23
1	BER ^1^: 0.4125	BER: 0.4125	BER: 0.4125	BER: 0.4	BER: 0.475	BER: 0.375	BER: 0.4125	BER: 0.4625	BER: 0.4875	BER: 0.4625
	MSE ^2^: 6.91 × 10^−8^	MSE: 6.91 × 10^−8^	MSE: 6.89 × 10^−8^	MSE: 8.24 × 10^−7^	MSE: 7.38 × 10^−8^	MSE: 1.10 × 10^−7^	MSE: 1.93 × 10^−7^	MSE: 3.13 × 10^−7^	MSE: 4.47 × 10^−7^	MSE: 5.78 × 10^−7^
5	BER: 0	BER: 0	BER: 0	BER: 0	BER: 0	BER: 0	BER: 0.0875	BER: 0.1625	BER: 0.35	BER: 0.425
	MSE: 1.08 × 10^−5^	MSE: 1.08 × 10^−5^	MSE: 1.08 × 10^−5^	MSE: 1.63 × 10^−5^	MSE: 1.09 × 10^−5^	MSE: 1.15 × 10^−5^	MSE: 1.22 × 10^−5^	MSE: 1.30 × 10^−5^	MSE: 1.40 × 10^−5^	MSE: 1.45 × 10^−5^
10	BER: 0	BER: 0	BER: 0	BER: 0	BER: 0	BER: 0	BER: 0	BER: 0.075	BER: 0.375	BER: 0.45
	MSE: 1.16 × 10^−5^	MSE: 1.16 × 10^−5^	MSE: 1.16 × 10^−5^	MSE: 2.08 × 10^−5^	MSE: 1.18 × 10^−5^	MSE: 1.26 × 10^−5^	MSE: 1.35 × 10^−5^	MSE: 1.48 × 10^−5^	MSE: 1.65 × 10^−5^	MSE: 1.72 × 10^−5^
15	BER: 0	BER: 0	BER: 0	BER: 0	BER: 0	BER: 0	BER: 0	BER: 0.1125	BER: 0.4375	BER: 0.45
	MSE: 1.14 × 10^−5^	MSE: 1.14 × 10^−5^	MSE: 1.14 × 10^−5^	MSE: 2.23 × 10^−5^	MSE: 1.16 × 10^−5^	MSE: 1.24 × 10^−5^	MSE: 1.35 × 10^−5^	MSE: 1.49 × 10^−5^	MSE: 1.70 × 10^−5^	MSE: 1.79 × 10^−5^
20	BER: 0	BER: 0	BER: 0	BER: 0	BER: 0	BER: 0	BER: 0	BER: 0.0625	BER: 0.4375	BER: 0.4875
	MSE: 1.24 × 10^−5^	MSE: 1.24 × 10^−5^	MSE: 1.24 × 10^−5^	MSE: 2.33 × 10^−5^	MSE: 1.26 × 10^−5^	MSE: 1.35 × 10^−5^	MSE: 1.46 × 10^−5^	MSE: 1.63 × 10^−5^	MSE: 1.90 × 10^−5^	MSE: 2.04 × 10^−5^
25	BER: 0	BER: 0	BER: 0	BER: 0	BER: 0	BER: 0	BER: 0.05	BER: 0.3125	BER: 0.4125	BER: 0.5375
	MSE: 1.20 × 10^−5^	MSE: 1.20 × 10^−5^	MSE: 1.20 × 10^−5^	MSE: 2.62 × 10^−5^	MSE: 1.23 × 10^−5^	MSE: 1.35 × 10^−5^	MSE: 1.49 × 10^−5^	MSE: 1.68 × 10^−5^	MSE: 1.97 × 10^−5^	MSE: 2.11 × 10^−5^
30	BER: 0	BER: 0	BER: 0	BER: 0	BER: 0	BER: 0	BER: 0	BER: 0.15	BER: 0.4375	BER: 0.4625
	MSE: 1.23 × 10^−5^	MSE: 1.23 × 10^−5^	MSE: 1.23 × 10^−5^	MSE: 3.02 × 10^−5^	MSE: 1.26 × 10^−5^	MSE: 1.37 × 10^−5^	MSE: 1.50 × 10^−5^	MSE: 1.71 × 10^−5^	MSE: 2.11 × 10^−5^	MSE: 2.30 × 10^−5^

^1^ Bit error rate of a single frame, ^2^ Mean square error of the encoded vs. original frame.

**Table 9:** Algorithm performance results for a 2160 × 3840 encoded video fragment with a 128 × 128 encoder trained for CRF 7 at a change in the BF with a change detection algorithm restoring the previous brightness factor value: 16-character-string decoding representation.

Frame #	CRF 7	CRF 7	CRF 7	CRF 7	CRF 7	CRF 7	CRF 7	CRF 7	CRF 7	CRF 7
	BF 5	BF 4	BF 3	BF 2	BF 1	BF −1	BF −2	BF −3	BF −4	BF −5
1	S: 2	S: 2	S: 3	S: 4	S: 4	S: 1	S: 1	S: 1	S: 1	S: 1
	M: 2	M: 2	M: 3	M: 4	M: 4	M: 1	M: 1	M: 1	M: 1	M: 1
5	S: 16	S: 16	S: 16	S: 16	S: 16	S: 1	S: 1	S: 1	S: 1	S: 1
	M: 16	M: 16	M: 16	M: 16	M: 16	M: 1	M: 1	M: 1	M: 1	M: 1
10	S: 16	S: 16	S: 16	S: 16	S: 16	S: 16	S: 1	S: 1	S: 1	S: 1
	M: 16	M: 16	M: 16	M: 16	M: 16	M: 1	M: 1	M: 1	M: 1	M: 1
15	S: 16	S: 16	S: 16	S: 16	S: 16	S: 16	S: 15	S: 1	S: 1	S: 1
	M: 16	M: 16	M: 16	M: 16	M: 16	M: 3	M: 1	M: 1	M: 1	M: 1
20	S: 16	S: 16	S: 16	S: 16	S: 16	S: 16	S: 16	S: 3	S: 1	S: 1
	M: 16	M: 16	M: 16	M: 16	M: 16	M: 16	M: 1	M: 1	M: 1	M: 1
25	S: 1	S: 16	S: 16	S: 16	S: 16	S: 16	S: 16	S: 16	S: 4	S: 1
	M: 16	M: 16	M: 16	M: 16	M: 16	M: 16	M: 5	M: 1	M: 1	M: 1
30	S: 1	S: 1	S: 5	S: 10	S: 11	S: 11	S: 8	S: 5	S: 4	S: 2
	M: 16	M: 16	M: 16	M: 16	M: 16	M: 16	M: 15	M: 1	M: 1	M: 1

**Table 10:** Algorithm performance results for a 2160 × 3840 encoded video fragment with a 128 × 128 encoder trained for CRF 7 when changing the video resolution: 16-character-string decoding representation.

Frame #	1080 × 1920	1940 × 3584	2032 × 3712	2160 × 3840	2288 × 3968	4320 × 7680	4608 × 8192
1	S: 1	S: 1	S: 1	S: 3	S: 1	S: 1	S: 1
	M: 1	M: 1	M: 1	M: 3	M: 1	M: 1	M: 1
5	S: 1	S: 5	S: 4	S: 16	S: 3	S: 5	S: 5
	M: 1	M: 5	M: 5	M: 16	M: 4	M: 4	M: 5
10	S: 1	S: 6	S: 8	S: 16	S: 6	S: 6	S: 6
	M: 1	M: 5	M: 5	M: 16	M: 5	M: 6	M: 6
15	S: 1	S: 7	S: 7	S: 16	S: 6	S: 8	S: 8
	M: 1	M: 7	M: 7	M: 16	M: 5	M: 6	M: 6
20	S: 1	S: 6	S: 7	S: 16	S: 6	S: 9	S: 8
	M: 1	M: 7	M: 7	M: 16	M: 6	M: 6	M: 6
25	S: 1	S: 5	S: 5	S: 16	S: 6	S: 6	S: 7
	M: 1	M: 7	M: 7	M: 16	M: 6	M: 6	M: 6
30	S: 1	S: 6	S: 6	S: 16	S: 6	S: 6	S: 6
	M: 1	M: 6	M: 6	M: 16	M: 6	M: 6	M: 6

**Table 11:** Algorithm performance results for a 2160 × 3840 encoded video fragment with a 128 × 128 encoder trained for CRF 7 when the image resolution is changed: representation of decoding (16 characters corresponding to 80 bits in the adopted numerical notion) in the forms of BER and MSE.

Frame #	1080 × 1920	1940 × 3584	2032 × 3712	2160 × 3840	2288 × 3968	4320 × 7680	4608 × 8192
1	BER: 0.55	BER: 0.55	BER: 0.55	BER: 0.4	BER: 0.55	BER: 0.55	BER: 0.55
	MSE: 2.08 × 10^−7^	MSE: 1.96 × 10^−7^	MSE: 1.98 × 10^−7^	MSE: 8.24 × 10^−7^	MSE: 5.63 × 10^−9^	MSE: 5.26 × 10^−9^	MSE: 5.22 × 10^−9^
5	BER: 0.4875	BER: 0.35	BER: 0.425	BER: 0	BER: 0.4	BER: 0.425	BER: 0.375
	MSE: 1.09 × 10^−5^	MSE: 1.07 × 10^−5^	MSE: 1.06 × 10^−5^	MSE: 1.63 × 10^−5^	MSE: 9.77 × 10^−6^	MSE: 9.64 × 10^−6^	MSE: 9.67 × 10^−6^
10	BER: 0.4625	BER: 0.3375	BER: 0.3	BER: 0	BER: 0.35	BER: 0.375	BER: 0.3625
	MSE: 1.52 × 10^−5^	MSE: 1.26 × 10^−5^	MSE: 1.26 × 10^−5^	MSE: 2.08 × 10^−5^	MSE: 1.21 × 10^−5^	MSE: 1.17 × 10^−5^	MSE: 1.17 × 10^−5^
15	BER: 0.5125	BER: 0.275	BER: 0.3375	BER: 0	BER: 0.3625	BER: 0.3375	BER: 0.3
	MSE: 1.41 × 10^−5^	MSE: 1.29 × 10^−5^	MSE: 1.29 × 10^−5^	MSE: 2.23 × 10^−5^	MSE: 1.22 × 10^−5^	MSE: 1.17 × 10^−5^	MSE: 1.17 × 10^−5^
20	BER: 0.5125	BER: 0.3	BER: 0.3375	BER: 0	BER: 0.3625	BER: 0.2625	BER: 0.3125
	MSE: 1.65 × 10^−5^	MSE: 1.43 × 10^−5^	MSE: 1.42 × 10^−5^	MSE: 2.33 × 10^−5^	MSE: 1.37 × 10^−5^	MSE: 1.31 × 10^−5^	MSE: 1.31 × 10^−5^
25	BER: 0.5125	BER: 0.3125	BER: 0.375	BER: 0	BER: 0.375	BER: 0.3875	BER: 0.35
	MSE: 1.79 × 10^−5^	MSE: 1.46 × 10^−5^	MSE: 1.44 × 10^−5^	MSE: 2.62 × 10^−5^	MSE: 1.38 × 10^−5^	MSE: 1.30 × 10^−5^	MSE: 1.30 × 10^−5^
30	BER: 0.5375	BER: 0.3	BER: 0.3625	BER: 0	BER: 0.3625	BER: 0.3875	BER: 0.3625
	MSE: 2.30 × 10^−5^	MSE: 1.64 × 10^−5^	MSE: 1.61 × 10^−5^	MSE: 3.02 × 10^−5^	MSE: 1.54 × 10^−5^	MSE: 1.38 × 10^−5^	MSE: 1.38 × 10^−5^

**Table 12:** Method comparison.

Type	Method 1 Zhou et al. [15]	Method 2 Gaj et al. [16]	Method 3 Liu et al. [43]	Proposed Method
Average PSNR (dB)	47.519	46.415	45.462	42.617
Capacity (bits/frame size)	100 bits/ 416 × 240	100 bits/ 416 × 240	100 bits/ 416 × 240	80 bits/ 128 × 128
Time for 20 frames of size 416 × 240 (ms) (Embedding time/Extraction time/Hardware)	32.478/ 5.622/ 3.30 GHz CPU, 4 GB RAM	36.855/ 5.048/ 3.30 GHz CPU, 4 GB RAM	34.058/ 5.997/ 3.30 GHz CPU, 4 GB RAM	34,457.92/3759.72/ Geforce 1080Ti GTX 11GB, 32 GB RAM
